# Optimization of Erythrocyte Lysis Protocols for Robust and Reproducible T Cell Analysis in Murine Peripheral Blood, Spleen, Lung, and Bone Marrow

**DOI:** 10.1155/jimr/3335332

**Published:** 2026-04-07

**Authors:** Wei-Qian Bao, Meng-Lin Jiang, Nan-Jie Zhou, Li-Rong Lian, Shu-qin Li, Bi-qing Huang, Tu-Liang Liang, Gang-Yuan Ma, Cui-Fen Zhang, Ying Chen, Xiao Shu, Liang Liu, Hu-Dan Pan, Run-Ze Li

**Affiliations:** ^1^ The Second Clinical Medical College, Guangzhou University of Chinese Medicine, Guangzhou, 510006, Guangdong, China, gzucm.edu.cn; ^2^ State Key Laboratory of Oncology in South China, Guangdong Provincial Clinical Research Center for Cancer, Sun Yat-sen University Cancer Center, Guangzhou, 510060, Guangdong, China, sysucc.org.cn; ^3^ Shenzhen Traditional Chinese Medicine Hospital, Shenzhen, 518000, Guangdong, China, szszyy.cn; ^4^ Department of Drug and Vaccine Research, Guangzhou Laboratory, Guangzhou, 510005, Guangdong, China; ^5^ State Key Laboratory of Traditional Chinese Medicine Syndrome, The Second Affiliated Hospital of Guangzhou University of Chinese Medicine (Guangdong Provincial Hospital of Chinese Medicine), Guangzhou, 510006, Guangdong, China, gzucm.edu.cn; ^6^ Traditional Chinese Medicine Immune Metabolism and Microecology Technology Development Platform, Chinese Medicine Guangdong Laboratory, Zhuhai, 519031, Guangdong, China

**Keywords:** erythrocyte lysis, flow cytometry, murine models, T cell analysis, tissue-specific protocols

## Abstract

Flow cytometry analysis of T lymphocytes in murine peripheral blood, spleen, lung, and bone marrow is critical for immune studies. However, inconsistent erythrocyte lysis remains a major challenge—incomplete lysis obscures lymphocyte detection, while excessive lysis damages T cells. Moreover, the variable performance of commercial lysis buffers across tissues and protocols complicates reproducibility. To address these challenges, we first established a lysis buffer that provides a relatively balanced effect on erythrocyte lysis and lymphocyte protection by systematically comparing BD, BioLegend, and Biosharp buffers in murine peripheral blood. Given the high content of red blood cells (RBCs) in peripheral blood, we used it as a screening model to identify a buffer that effectively lysed RBCs while maintaining lymphocyte integrity. The BD lysing solution demonstrated superior performance, achieving effective erythrocyte removal with 41.40% CD3^+^ cell of the lymphocyte population at 1‐min lysis (compared to BioLegend: 38.70% and Biosharp: 36.90% at their recommended durations). This buffer was then used as the unified buffer for subsequent multitissue optimization, ensuring a balanced effect across different tissue types. Building on this foundation, we developed a standardized framework, which resolves tissue‐specific barriers. Spleen: Balanced erythrocyte clearance was achieved using 150 μL/million cells, yielding 67.23% ± 8.21% CD3^+^ lymphocytes. CD4^+^ and CD8^+^ T cell subsets constituted 58.50% ± 0.85% and 36.63% ± 0.85% of CD3^+^ cells, respectively (CD4/CD8 ratio: 1.60 ± 0.60). Lung: Adaptive processing volumes (30–150 μL/million cells) accommodated variable dissociation yields (1–5 × 10^6^ cells/sample). CD45^+^ cells represented 42.87% ± 6.05% of live cells, with CD3^+^ cells dominating the lymphocyte compartment (96.57% ± 1.35%). CD4^+^ (58.50% ± 1.44%) and CD8^+^ (39.23 ± 1.12%) T cell proportions mirrored splenic distribution. Bone marrow: Erythrocyte depletion at 33.3 μL/million cells preserved rare lymphocytes (6.53% ± 0.30% of total cells). CD3^+^ cells comprised 21.80% ± 3.26% of CD45^+^ lymphocytes, exhibiting distinct subset ratios (CD4^+^: 57.34% ± 4.60%; CD8^+^: 50.50% ± 2.78%). Collectively, these protocols enable robust, operator‐independent T cell analysis across murine immune compartments, providing a universal standard for translational immunology studies.

**Summary**

• A standardized framework for cross‐tissue T cell analysis was established, balancing highly efficient erythrocyte lysis with superior T cell preservation. This novel approach introduces a ratio‐driven and volume‐adjusted lysis strategy that dynamically tailors buffer volume to nucleated cell count, overcoming reproducibility challenges caused by sample‐to‐sample cellularity variations. It ensures maximal T cell viability while effectively clearing erythrocytes.
• An objective cell‐to‐buffer ratio replaces subjective timing, markedly improving procedural consistency. By shifting the critical control parameter from an empirical time window (e.g., “3–5 min”) to a precise and measurable metric, this method minimizes operator‐dependent variability and enhances interlaboratory reproducibility.
• Robust immunophenotyping results were validated across tissues with minimal sample consumption. The framework yielded highly consistent data (e.g., 67.23% CD3^+^ in spleen and 96.57% in lung lymphocytes) while using only a small fraction of the original sample for optimization. This conserves precious primary material, empowering the systematic optimization of cell‐type‐specific lysis conditions for a wide array of immune cells.

## 1. Introduction

In‐depth exploration of the immune system is essential for understanding disease etiology, developing treatment strategies, and advancing immunology. Flow cytometry, a versatile cell analysis technique, has become crucial for investigating the immune system [1–3]. In mouse models, flow cytometry of peripheral blood, spleen, lung, and bone marrow is indispensable for understanding immune system intricacies [4]. However, literature systematically documenting flow cytometric methods across these tissues is scarce. Systematic assessment of T lymphocyte changes in peripheral blood, spleen, lung, and bone marrow provides comprehensive insights into T lymphocyte development, maturation, activation, and tissue residency. This approach enhances understanding of immune diseases by analyzing overall and tissue‐specific immune states [4, 5]. Lung tissue, a primary barrier tissue [6], is included to explore tissue residency and tissue‐specific T cells. Flow cytometry of these samples presents challenges. Mouse lymph nodes are small and difficult to distinguish from adipose tissue, complicating precise surgical collection [7]. Bone marrow, the site of hematopoiesis, contains diverse cell types, including hematopoietic stem cells, stromal cells, and various blood cells, complicating lymphocyte analysis [8]. Similarly, analyzing lymphocytes in mouse lung tissue is challenging due to its cellular diversity and the unsuitable nature of conventional tissue grinding methods [9].

In flow cytometry, red blood cell (RBC) lysis is a crucial step in sample preparation, especially when analyzing samples rich in RBCs, such as peripheral blood, spleen, bone marrow, and lung tissue. Common challenges include inadequate erythrocyte lysis [10], leading to poor separation between lymphocytes and RBCs or debris, and excessive lysis, resulting in damage to cell counts [11, 12], morphology [13], and antigenic epitopes [13]. Insufficient lysis is particularly prevalent in peripheral blood samples. Additionally, the varying instructions for erythrocyte lysis solutions among different brands require researchers to determine optimal conditions through extensive experimentation. The process of RBC lysis can significantly influence flow cytometric results. Einwallner et al. [14] demonstrated significant differences in the percentage of immature cells detected when using four different erythrocyte lysis solutions on peripheral blood samples from leukemia patients, highlighting the importance of standardized protocols. Similarly, Plank et al. [15] found that varying efficiencies in erythrocyte lysis solutions led to specific biases in leukocyte subpopulation quantification.

Numerous brands of RBC lysis solutions are available, each with different instructions regarding temperature, time, volume, and termination conditions. For instance, BD FACS lysing solution 10x concentrate requires dilution with room temperature (20–25°C) deionized water at a tenfold dilution, but lacks specific guidance on the amount of lysis solution needed for different samples or termination procedures [16]. BioLegend RBC lysis buffer (10x) recommends diluting to a 1 × working concentration with deionized water, adding 100 μL of whole blood to 2.0 mL of 1 × RBC lysis buffer, gently vortexing, incubating at room temperature in the dark for 10–15 min, centrifuging at 350 × *g* for 5 min, and proceeding with flow cytometry [17]. However, it does not specify conditions for mouse peripheral blood or termination steps. In our laboratory observations, terminating erythrocyte lysis is crucial to protect lymphocytes from excessive damage. Biosharp and Beyotime brands recommend adding 6–10 times the cell volume of RBC lysis buffer to fresh anticoagulated blood samples, gently mixing, incubating at room temperature or 4°C for 1–2 min, and centrifuging at 400–500 × *g* for 5 min at 4°C for optimal results. For small samples, the supernatant can be omitted, and RBC lysis buffer can be added directly at a tenfold volume of blood, incubated at room temperature or 4°C for 4–5 min [18]. However, we found that these instructions often did not yield satisfactory results. There is considerable variation in erythrocyte lysis conditions used in different studies. For example, Moraes‐Vieira et al. [19] and Krishnarajah et al. [4] used BioLegend erythrocyte lysis buffer, incubating at room temperature for 3 and 5 min, respectively, without specifying the volume of lysis solution or termination conditions. Zhu et al. [20] incubated at room temperature for 10 min, while Skordos et al. [10] used Gibco lysis buffer, incubating 5 mL at room temperature for 3 min to lyse mouse blood samples.

To address these limitations, we developed a standardized framework to optimize erythrocyte lysis across murine blood, spleen, lung, and bone marrow. By integrating dynamic volumetric scaling (tissue‐specific buffer‐to‐cell ratios), time‐independent lysis control, and erythrocyte‐exclusion counting, this study resolves the trade‐off between erythrocyte clearance and lymphocyte preservation inherent in conventional protocols. Furthermore, we systematically evaluated commercial lysis buffers (BD, BioLegend, and Biosharp) to establish a reagent‐agnostic optimization strategy. These innovations collectively provide a unified methodology for robust T cell analysis, addressing reproducibility challenges in multi‐tissue immunology studies.

## 2. Materials and Methods

### 2.1. Mouse Strains

C57BL/6J mice (6–8 weeks old, female, SPF grade) were obtained from the Animal Experimental Center of the Guangdong Provincial Hospital of Traditional Chinese Medicine and accommodated by dedicated personnel in a barrier environment. Approval for all experimental procedures in this study was granted by the Animal Ethics Committee of the Guangdong Provincial Hospital of Traditional Chinese Medicine. All surgical procedures were performed under tribromoethanol anesthesia (250 mg/kg, intraperitoneal injection) following institutional guidelines. Anesthesia depth was verified by loss of pedal withdrawal reflex and respiratory rate monitoring (maintained at 80–120 breaths/min). Terminal blood collection was achieved via cardiac puncture under deep anesthesia, followed by cervical dislocation as secondary euthanasia method.

For each experimental condition, three mice were used as biological replicates, and the experimental operation was conducted following a “one operator per mouse” design during the tissue harvesting phase: each operator was exclusively responsible for harvesting four distinct murine tissues (peripheral blood, spleen, lung, and bone marrow) from one designated mouse. Subsequent to harvesting, the operator proceeded with tissue processing (including the preparation of single‐cell suspensions from the spleen, lung, and bone marrow) and cell counting for the tissues of their assigned mouse. After completing these steps for all mice, samples of the same tissue type from different mice were pooled. Postpooling, the workflow was streamlined with a clear division of labor among the three operators: one operator handled the pooled peripheral blood and spleen samples, another was responsible for the pooled lung samples, and the third processed the pooled bone marrow samples.

The rationale for this two‐phase design (i.e., “one operator per mouse” during harvesting/initial processing, followed by operator‐specific tissue‐type processing postpooling) is as follows: First, given the large sample size and the need for multiple erythrocyte lysis protocol comparisons, this design avoids operator overload and ensures consistent sample handling quality. Second, as flow cytometry–based T cell analysis relies critically on fresh samples to maintain cell phenotype integrity and data accuracy, delayed processing can cause cell damage or phenotypic alterations. Assigning one operator to one mouse enables synchronized harvesting and initial processing of the four tissues from the same individual, preventing delays from cross‐handling multiple mice to preserve sample freshness and avoiding batch effects (e.g., altered cell viability or subset proportions) from sequential processing of samples from a single mouse. Third, since each tissue requires lysis condition optimization, processing multiple mice by one or multiple operators may introduce confounding interoperator variability (e.g., in handling speed, lysis timing precision, or suspension uniformity). The “one operator per mouse” design ensures consistent handling of all steps for a single mouse, minimizing such variability.

Notably, for the practical application of the optimized erythrocyte lysis protocol established in this study, the streamlined postpooling workflow (with dedicated operators for specific tissue types) is recommended for routine use. This workflow eliminates the need for comparing multiple lysis schemes and leverages the optimized parameters identified in the study. By assigning specific tissue types to individual operators, it not only reduces operator dependence but also significantly improves experimental efficiency while ensuring the reproducibility of results.

### 2.2. Main Reagents and Materials

All analytical‐grade reagents, antibodies, and consumables used in this study were obtained from commercial sources. Detailed information, including supplier, catalog number, working dilution, and buffer formulation, is summarized in Table 1 (buffer composition), Table 2 (full reagent and material list), and Table 3 (monoclonal antibody usage parameters).

**Table 1:** Composition of buffers for erythrocyte lysis and cell staining.

Buffer type	Composition (units included in description)
PEB buffer	PBS + 2% FBS + 1mM EDTA or PBS + 0.5% BSA + 1 mM EDTA
Cell staining buffer	PBS + 2% FBS
1× lysing solution	Lysing solution 10× concentrate diluted 10× in purified deionized water

**Table 2:** Main reagents and materials.

Reagent or resource	Source	Identifier
Fixable Viability Stain 620	BD	Cat#564996
Ghost Dye Violet 540 fixable viability dye	TONBO	Cat#72086
Anti‐CD45 antibody	BD	Cat#552848
Anti‐CD3 antibody	BioLegend	Cat#100234
Anti‐CD3 antibody	BioLegend	Cat#100204
Anti‐CD4 antibody	BD	Cat#553051
Anti‐CD4 antibody	BioLegend	Cat#100456
Anti‐CD8a antibody	BioLegend	Cat#100706
Anti‐CD8a antibody	BioLegend	Cat#100712
Lysing solution 10x concentrate	BD	Cat#349202
RBC lysis buffer (10x)	BioLegend	Cat#420301
Red blood cell lysis buffer	Biosharp	Cat#BL503B
3% acetic acid with methylene blue	Stemcell	Cat#07060
Fetal bovine serum (FBS)	ExCell	Cat#FSP500
Albumin from bovine serum	Macklin	Cat#B917420‐200g
Phosphate‐buffer saline (PBS)	Solarbio	Cat#G0002
MACS tissue storage solution	Miltenyi	Cat#130‐100‐008
Lung dissociation kit, mouse	Miltenyi	Cat#130‐095‐927
gentleMACS 25C Tubes C	Miltenyi	Cat#130‐093‐237
70 μm cell strainers	Biosharp	Cat#BS‐70‐XBS‐N1
Heparin sodium anticoagulant tube 1.5 mL	Solarbio	Cat#YA1471
Isoflurane anesthetic	RWD	Cat#R510‐22‐10

**Table 3:** Usage of monoclonal antibodies.

Antigen	Clone	Fluorochrome	Dilution	Surface/intracellular
Fixable Viability Stain 620	—	FVS620	1:1000	Surface
Ghost Dye Violet 540 fixable viability dye	—	Violet 540	1:5000	Surface
CD45	30‐F11	PE‐Cy7	3:1000	Surface
CD3	17A2	BV510	1:200	Surface
CD3	17A2	FITC	1:500	Surface
CD4	RM4‐5	APC	1:800	Surface
CD4	GK1.5	PE/Dazzle 594	1:800	Surface
CD8a	53‐6.7	FITC	1:500	Surface
CD8a	53‐6.7	APC	1:800	Surface

### 2.3. Flow Cytometric Analysis of T Lymphocytes in Mouse Peripheral Blood

Peripheral blood was collected from anesthetized mice using heparin sodium anticoagulant. A 50 μL aliquot of blood was transferred to prechilled microcentrifuge tubes for immediate processing. RBCs were lysed with 500 µL of 1 × BD lysis solution (Table 1 and 2), immediately vortexed, and then incubated for 1 min at room temperature in the dark. The reaction was terminated with 500 µL of precooled cell staining buffer, followed by centrifugation at 350 × *g* for 5 min at 4°C. The cell pellet was washed twice with PBS (centrifugation at 300 × *g*, 3 min, 4°C), retaining approximately 100 µL supernatant after the final wash. Viability staining was performed using a diluted viability dye (Table 1 and 2) for 10–15 min at room temperature in the dark, followed by centrifugation and an additional wash step. Surface antibody staining was conducted using preoptimized antibody cocktails (Table 3) incubated on ice for 20 min in the dark. Cells were washed twice with PBS and resuspended in 800 µL cell staining buffer for acquisition. A minimum of 1 × 10^4^ events per sample were recorded on the flow cytometer. A detailed and step‐by‐step standard operating procedure (SOP) is provided in Supporting Information 1 to ensure reproducibility and minimize interoperator variability.

### 2.4. Flow Cytometric Analysis of T Lymphocytes in Mouse Spleen

Splenocyte suspension was prepared using a novel syringe‐based isolation technique. Carefully excise the spleen, rinse in ice‐cold PBS, and transfer to a culture dish containing 2 mL of ice‐cold PBS. The spleen was held with forceps while PBS was injected multiple times using a 1 mL syringe until the tissue color changed from dark red to light red. The resulting cell suspension (~3 mL total volume) was collected and mixed thoroughly. Cell counting was performed using 3% acetic acid with methylene blue (Table 2), and 1 × 10^6^ cells were transferred to a 1.5 mL tube for flow cytometry analysis. The cell pellet was washed once with PBS (centrifugation at 350 × *g* for 5 min at 4°C), ensuring complete supernatant removal to avoid interference with subsequent steps. After the addition of 100–200 µL of 1 × BD lysis solution (Table 1 and 2), the cells were immediately vortexed and incubated for 1 min at room temperature in the dark. The reaction was terminated with 1 mL of precooled cell staining buffer, followed by centrifugation. Two additional washes were performed with PBS (centrifugation at 300 × *g* for 3 min at 4°C), retaining approximately 100 µL supernatant after the final wash. Flow cytometry analysis was conducted as described in Section 2.3, recording a minimum of 1 × 10^4^ events per sample. To enhance consistency across experiments, a detailed SOP outlining each technical maneuver is available in Supporting Information 1.

### 2.5. Flow Cytometric Analysis of T Lymphocytes in Mouse Lung

Prior to tissue removal, 10 mL of cold PBS was injected into the right ventricle until lungs turned white to minimize blood lymphocyte contamination. Lungs were separated, rinsed, and placed in cold PBS on ice. The overall workflow (tissue dissociation, single‐cell preparation, and flow cytometry) referred to D’Agostino et al. [21] and Rottmann et al. [22], with a key modification: instead of their self‐prepared digestion solutions (collagenase combinations [21]; collagenase P/II + DNase I [22]), we used a commercial lung dissociation kit (mouse), simplifying procedures, reducing manual variability, and ensuring efficient dissociation and high cell viability. Lung tissue was dissociated into single‐cell suspension using the kit (Table 2) and gentleMACS Octo Dissociator. The suspension was filtered through a 70 μm strainer, rinsed with 2.5 mL 1 × Buffer S, centrifuged (300 g, 10 min), and resuspended in 3 mL staining buffer (Table 2). Cell counting was performed with 3% acetic acid‐methylene blue (Table 2), and 3 × 10^6^ cells/tube were transferred for analysis. The pellet was washed (350 × *g*, 4°C, 5 min), lysed with 150 µL 1 × BD lysis solution (Table 1 and 2) for 1 min at RT in the dark, terminated with 1 mL precooled staining buffer, and washed twice (300 × g, 4°C, 3 min). Flow cytometry was conducted as Section 2.3, recording ≥1 × 10^5^ events/sample. A detailed protocol is provided in the Supporting Information.

### 2.6. Flow Cytometric Analysis of T Lymphocytes in Mouse Bone Marrow

The bone marrow collection and single‐cell suspension preparation referred to Haubruck et al. [5], and flow cytometry workflow referred to Olexen et al. [23], with two key innovations in cell counting. The femur was isolated, cleaned, and both ends removed; marrow was flushed with cold PBS until the bone turned white (optimized from Haubruck et al. [5], emphasizing thorough collection). The cell suspension was filtered through a 70 μm strainer and rinsed with PBS to 5 mL. Cell counting was performed using 3% acetic acid with methylene blue (Table 2) at this step; in contrast, Haubruck et al. [5] and Olexen et al. [23] use trypan blue and count after RBC lysis. These innovations (3% acetic acid–methylene blue counting and prelysis counting) are key methodological advances of this study, enabling accurate quantification of initial marrow yield, avoiding lysis‐induced cell loss underestimation, and ensuring consistent cell input (3 × 10^6^ cells/tube) for flow cytometry. The cell pellet was washed with PBS (350 × *g*, 4°C, 5 min), lysed with 100 µL of 1 × BD lysis solution (Table 1 and 2) for 1 min at RT in the dark, terminated with 1 mL precooled staining buffer, and washed twice (300 × g, 4°C, 3 min). Flow cytometry was conducted as Section 2.3, recording ≥1 × 10^4^ events/sample. A detailed protocol is provided in the Supporting Information.

### 2.7. Statistical Analysis

Data are presented as mean ± standard deviation (SD). Descriptive statistics (mean and SD) were used to summarize the central tendency and dispersion of the data from the exact number of replicates indicated in each figure legend. No inferential statistical tests (e.g., *t*‐tests and ANOVA) were applied, as the study aimed to establish an optimized protocol and report its performance metrics rather than test differences between predefined experimental groups.

## 3. Results

### 3.1. Stable and Efficient Lysis Protocol for 50 μL Mouse Peripheral Blood Samples Using 500 μL BD Lysing Solution at Room Temperature for 1 min

First, for BD lysing solution, since specific lysis conditions are not mentioned in the manual, we referred to the protocols of Biosharp and Beyotime. For small blood samples, add 10 times the volume of erythrocyte lysate and lyse at room temperature or 4°C for 4–5 min. To terminate lysis and minimize lymphocyte damage, our experience suggests adding an equal volume of cell staining buffer. Without termination, lymphocytes may undergo further damage during centrifugation.

For 50 μL samples of mouse peripheral blood, we added either 500 μL or 1 mL of 1 × BD erythrocyte lysate and performed lysis for 3, 5, and 10 min at room temperature. Lysis was terminated by adding 500 μL of cold cell staining buffer (Table 1) and centrifuging for 5 min at 4°C. With 500 μL of BD lysate, clear lymphocyte clusters were observed after 3 min of lysis, smaller clusters after 5 min, and increased cellular debris after 10 min (Figures 1C and 2A). The percentages of lymphocytes at 500 μL volume for 3, 5, and 10 min of lysis were 3.47%, 0.94%, and 4.54%, respectively (Figure 1C), far below the values reported for healthy mice. Considering the potential damage from prolonged lysis, we evaluated erythrocyte lysis at room temperature for 1 and 2 min and at 4°C for 1 min. Considering the potential damage from prolonged lysis, we evaluated erythrocyte lysis at room temperature for 1 and 2 min and at 4°C for 1 min. The lysis condition of 1 min at room temperature is the most ideal (Figure 1D). This condition not only effectively lyses RBCs and reduces RBC debris interference but also maximally preserves the integrity and activity of lymphocytes, resulting in the highest recovery rate of CD3^+^ lymphocytes (42.20%). In contrast, 2 min of lysis at RT leads to lymphocyte damage, while 1 min of lysis at 4°C results in incomplete lysis. Therefore, neither of these conditions is the optimal choice.

Figure 1Optimized 1‐min room temperature lysis with BD solution maximizes lymphocyte integrity in murine peripheral blood. (A) Schematic workflow for murine tissue sampling and processing. (B) Experimental design for erythrocyte lysis condition screening. Both Subfigures (A, B) are created in BioRender. Pau, C. (2026) https://BioRender.com/q1x163p. (C, D) Condition screening. (C) Extended lysis duration (>3 min) with BD solution induces severe lymphocyte loss (3‐min: 3.47%; 5‐min: 0.94%; 10‐min: 4.54%) and debris accumulation. (D) 1‐min RT lysis achieves superior CD3^+^ T cell recovery (42.2%) versus 2‐min RT (damaged lymphocytes) or 1‐min 4°C (incomplete lysis), with 90.26% erythrocyte removal (FSCA/SSC‐A gating). Data in Subfigures (C, D) are from technical replicates (*n* = 3 per condition) derived from one pooled blood sample (from three female C57BL/6J mice) and are representative of two independent experiments, demonstrating the approach’s utility for high‐throughput parameter screening. (E–G) Protocol validation. (E) Representative plots after 500 μL BD lysis at RT for 1 min. (F) Gating strategy for lymphocyte and T cell subsets. (G) Summary data showing preservation of T cell subsets: Lymphocytes 11.13% ± 2.50%, CD3^+^ 43.40% ± 3.27%, CD4^+^/CD8^+^ ratio 1.56 ± 0.72, consistent with the literature [21, 22]. Data in Subfigures (E–G) are from biologically independent mice (*n* = 3), processed individually to assess protocol robustness across individuals.

**Figure:**
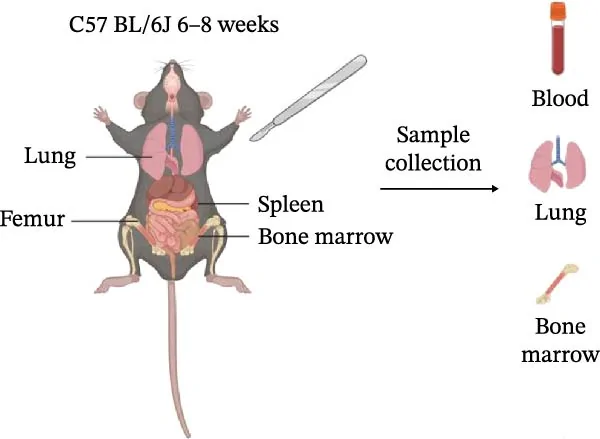
(A)

**Figure:**
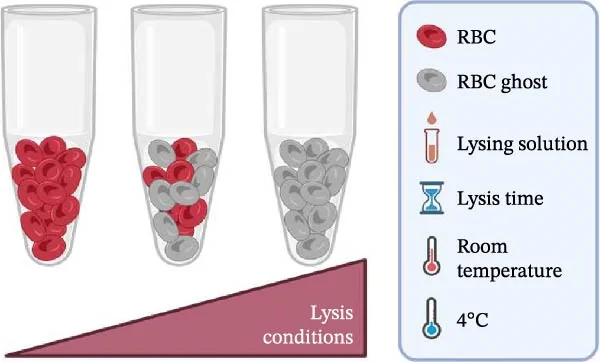
(B)

**Figure:**
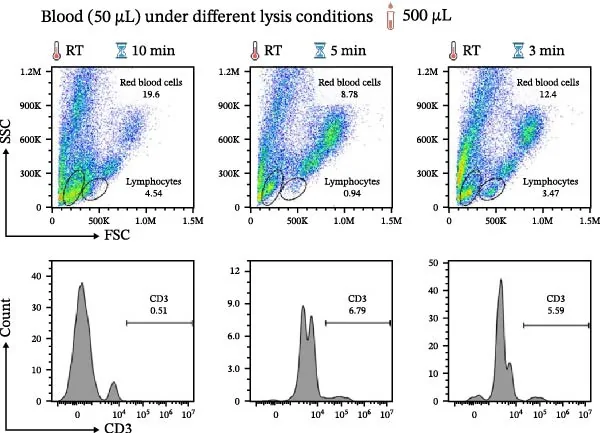
(C)

**Figure:**
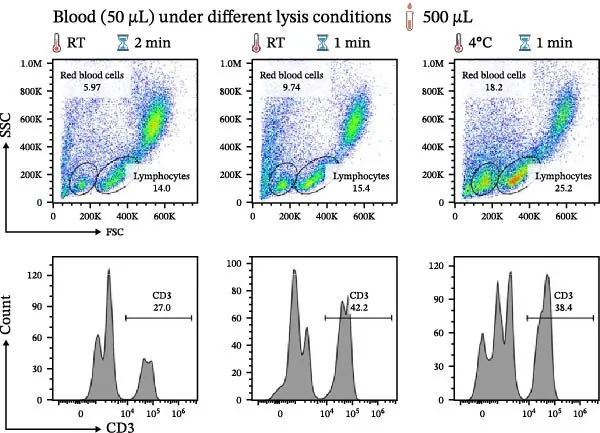
(D)

**Figure:**
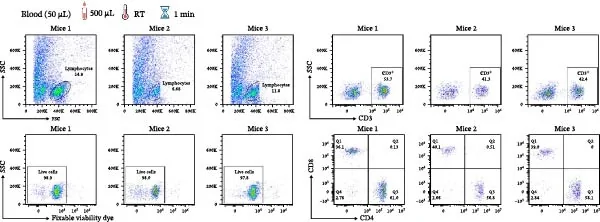
(E)

**Figure:**
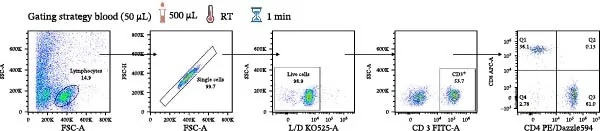
(F)

**Figure:**
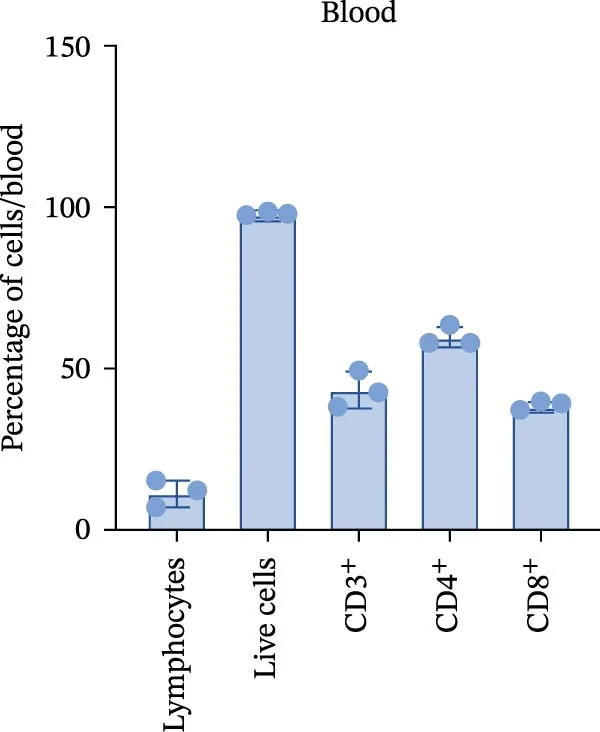
(G)

Figure 2BD lysis achieves maximal efficiency with minimal resource burden versus competitors. (A) BD protocol scalability. A 1 mL volume with 3‐min lysis maintains CD3^+^ yield (20.2%), relative to the BD standard 500 μL/1‐min protocol (full validation in Figure 1, 41.4% measured in the same experimental batch). (B) BioLegend protocol. Imposes a 10x duration burden for equivalent output: 2 mL/10‐min lysis delivers comparable debris control (6.06% vs. BD 10.3%) and CD3^+^ yield (38.7% vs. the aforementioned BD standard protocol, 41.4%), necessitating an increase in flow instrumentation threshold settings to mitigate interference from the elevated cellular debris. (C) Biosharp protocol. Exhibits significant batch variability: Lot#1 (3 mL/4°C/3‐min lysis) preserves lymphocytes, whereas Lot#2 (500 μL protocol) fails regardless of duration, underscoring the need for pre‐validation. Data in (A–C) are derived from a single pooled blood sample collected from three female C57BL/6J mice, which was aliquoted into 50 μL technical replicates (*n* = 3 per lysis condition) for high‐throughput comparative screening. Comparative lysis was performed using BD (#349202), BioLegend (#420301), and Biosharp (#BL503B, Lot#1: 221181; Lot#2: 23222365) reagents. Flow cytometry was conducted on a Beckman CytoFlex S, with the FSC‐A threshold optimized for each condition. The validated stable performance of this BD standard protocol, based on independent biological replicates, is presented in Figure 1, with a CD3^+^ yield of 43.40% ± 3.27%; the 41.4% value in this figure is the technical replicate measurement of the same protocol in the same experimental batch, which is within the normal error range of the validated stable value.

**Figure:**
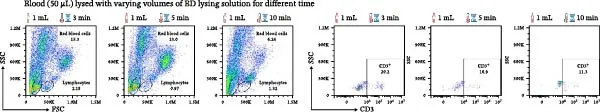
(A)

**Figure:**
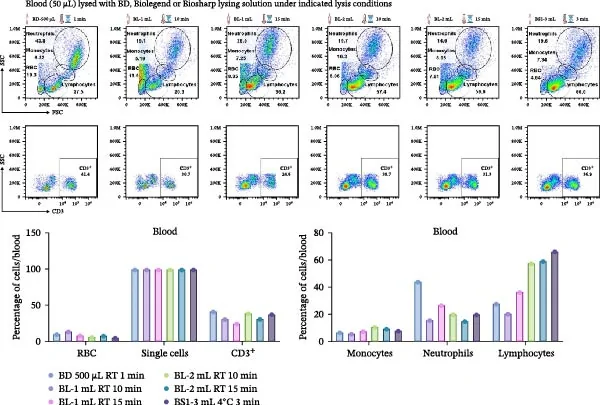
(B)

**Figure:**
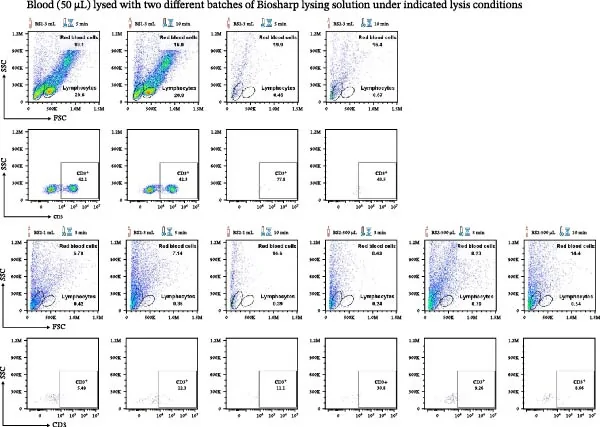
(C)

Based on our findings, we recommend the following protocol for 50 μL mouse peripheral blood samples: Add 500 μL of 1 × BD lysing solution, mix thoroughly, and lyse in the dark at room temperature for 1 min. Immediately add 500 μL of precooled staining buffer (Table 1) to terminate the reaction, followed by centrifugation at 350 × *g* at 4°C for 5 min. Using this protocol, the proportion of lymphocytes in the peripheral blood of three mice was 11.13% ± 2.50%, with CD3^+^ lymphocytes constituting 43.40% ± 3.27% of the lymphocyte population. The percentages of CD4^+^ and CD8^+^ T cells were 59.83% ± 1.79% and 38.40% ± 0.60%, respectively, resulting in a CD4 to CD8 ratio of 1.56 ± 0.72 (Figure 1E–G). These results are similar to the reported percentages of 55.90% for CD4^+^ T cells and 42.70% for CD8^+^ T cells among T cells [24]. The CD4 to CD8 ratio also aligns with findings reported by Pinchuk and Filipov [25]. To evaluate the intragroup consistency of the optimized protocol, we calculated the coefficient of variation (CV, defined as (SD/mean) × 100%) of CD3^+^, CD4^+^, and CD8^+^ T cell proportions. The CV values of peripheral blood T cell subsets were relatively low (Table 4), indicating good intragroup consistency of the results generated by the optimized protocol with three biological replicates.

**Table 4:** Comparison of coefficient of variation (CV) of CD3^+^, CD4^+^, and CD8^+^ T cell proportions between our optimized protocol and traditional methods across four murine tissues.

Tissue type	T cell subset	Mean ± SD (%) (our protocol)	CV value of our protocol (%)	CV value of traditional methods (%)	References
Peripheral blood	CD3^+^	43.40 ± 3.27	7.53	66.86	Litvinova et al. [26] (*n* = 11)
	CD4^+^	59.83 ± 1.79	2.99	–	No literature data
	CD8^+^	38.40 ± 0.60	1.56	20.74	Jiang et al. [27] (*n* = 152)
Spleen	CD3^+^	67.23 ± 8.21	12.21	–	No literature data
	CD4^+^	58.50 ± 0.85	1.45	23.53–43.75	Magee et al. [28] (*n* = 8–10)
	CD8^+^	36.63 ± 0.85	2.32	10.00–40.00	Magee et al. [28] (*n* = 8–10)
Lung	CD3^+^	96.57 ± 1.35	1.40	–	No literature data
	CD4^+^	58.50 ± 1.44	2.46	4.81	Liu et al. [29] (*n* = not specified)
	CD8^+^	39.23 ± 1.12	2.85	–	No literature data
Bone marrow	CD3^+^	21.80 ± 3.26	14.95	–	No literature data
	CD4^+^	31.83 ± 2.48	8.41	21.0	Berger et al. [30] (*n* = 10)
	CD8^+^	50.50 ± 2.78	5.50	19.2	Berger et al. [30] (*n* = 10)

*Note*: CD4^+^ proportion refers to the percentage of CD4^+^ cells within CD3^+^ cells; CV was calculated as (SD/mean) × 100%; *n* = 3 per group for our protocol. “–” indicates no literature data available for the corresponding tissue/subset in the “CV value of traditional methods (%)” column. For Litvinova et al. [26] and Berger et al. [30] (data presented as mean ± SE), SD was converted from SE before CV calculation; Magee et al. [28], Liu et al. [29], and Jiang et al. [27] data were presented as mean ± SD, and CV was calculated directly. Only the five provided literatures are included for traditional methods. “*n* = not specified” indicates sample size not reported in Liu et al. [29]. Jiang et al. [27] reports data from female BALB/c mice, while our protocol uses C57BL/6J mice and provides CD8^+^ T cell CV for peripheral blood for comparison. Higher CV values of CD3^+^ T cells in spleen and bone marrow (our protocol) may be associated with biological variability among individuals, technical differences in flow cytometry experiments, and individual sensitivity to lysis buffer.

Abbreviation: SD, standard deviation.

Given the widespread use of BioLegend RBC lysis buffer (Table 2), we investigated four erythrocyte lysis conditions using BioLegend RBC lysis buffer and compared these with the recommended BD lysis protocol as previously mentioned. For 50 μL samples of mouse peripheral blood, we added either 1 or 2 mL of 1 × BioLegend erythrocyte lysing solution and performed lysis for 10 and 15 min at room temperature, followed by the addition of an equal volume of precooled staining buffer to stop the process, and centrifuged at 350 × *g* at 4°C for 5 min. The results indicated that lysis with 2 mL of BioLegend RBC lysis buffer for 10 min yielded the most distinct lymphocyte clusters, with 38.70% of lymphocytes being CD3^+^ (Figure 2B), which is within the range of 17.00%–48.60% reported in the literature. Compared to the BD lysis condition (500 μL for 1 min), the BioLegend protocol using 2 mL RBC lysis buffer for 10 min demonstrated a higher proportion of lymphocyte subpopulations with reduced lymphocyte damage. However, lymphocyte clusters exhibited closer proximity to cellular debris and erythrocytes under the BioLegend condition than under the recommended BD protocol (Figure 2B). When performing flow cytometry analysis (Beckman CytoFlex S), threshold settings were required to mitigate interference from the increased cellular debris observed with BioLegend. Although the population distinction was less pronounced compared to the BD lysis method described in this study, the BioLegend RBC lysis buffer remains a viable option for erythrocyte lysis in murine peripheral blood samples. Descriptions of the differential sensitivity of lymphocytes, neutrophils, and monocytes to different commercial lysis reagents and lysis conditions, with supporting data from Figure 2B (including gated granulocyte/macrophage populations, percentage annotations, and bar graphs summarizing quantitative results). Specifically, we highlighted the comprehensive performance of the BD 500 μL RT 1 min condition in preserving major immune cell subsets. Ultimately, we extended the application of BD lysis conditions to other experimental samples while acknowledging the general applicability of BioLegend lysis buffer for this purpose.

Additionally, we initially investigated erythrocyte lysis using the domestic brand Biosharp, which demonstrated mild yet effective lysis with 3 mL of lysing solution on ice for 3 min (Figure 2B), without significant damage to lymphocytes even when extending the duration to 5 and 10 min (Figure 2C). However, variability between different lot numbers was observed with Biosharp RBC lysis buffer. Further examination of another lot from the same catalog number showed that reducing the lysis volume to 500 μL and varying the time between 3, 5, and 10 min impeded the observation of lymphocyte subpopulations (Figure 2C). Therefore, we recommend conducting preliminary experiments to determine the optimal lysis conditions when using Biosharp RBC lysis buffer, especially when transitioning to a new lot number.

### 3.2. Reliable and Efficient Lysis of 1 × 10^6^ Mouse Splenocytes Using 150 μL BD Lysing Solution at Room Temperature for 1 min

The significant differences between spleen tissue and peripheral blood samples necessitated a reassessment of erythrocyte lysis conditions using BD brand lysis buffer, specifically tailored for spleen tissue. Initially, splenocyte suspensions were prepared using the repeated injection method (Figure 3A). The efficacy of erythrocyte lysis was evaluated by comparing flow cytometry results for samples treated in the dark at room temperature for 1 min with varying volumes of 1 × BD erythrocyte lysis solution, specifically 0, 30, 50, 100, 150, and 200 µL. Optimal results were observed with 100, 150, and 200 µL of lysis solution, where distinct lymphocyte clustering was evident. The percentages of CD3^+^ lymphocytes were 60.90%, 61.40%, and 65.6%, respectively (Figure 3B). Additionally, the proportions of CD4^+^ T cells within the CD3^+^ lymphocyte population were 44.90%, 46.30%, and 44.20%, respectively, while the proportions of CD8^+^ T cells were 27.20%, 28.90%, and 26.00% respectively (Figure 3B). The 150 μL volume achieves maximal cost‐efficacy balance—saving 25% reagent versus 200 μL protocol while maintaining equivalent CD3^+^ yield (61.40% versus 65.60%), critical for large‐scale studies.

Figure 3Ratio‐driven volumetric titration identifies 150 μL/million splenocytes under 1‐min RT lysis for optimal splenocyte processing with minimal residual erythrocytes. (A) Mechanical dissociation protocol for murine splenocytes. Created in BioRender. Pau, C. (2026) https://BioRender.com/mpez99o. (B) Titration screening. A 150 μL lysate per million cells achieves peak CD3^+^ recovery (61.4%) versus suboptimal volumes (30 μL: incomplete lysis; 200 μL: viability loss), eliminating 67.4% erythrocytes (FSC‐A/SSC‐A gate). Data are from technical replicates (*n* = 3 per condition) derived from one pooled splenocyte sample (from three female C57BL/6J mice). (C–E) Protocol validation. (C) Representative plots after 150 μL BD lysis at RT for 1 min. (D) Gating strategy for lymphocyte and T cell subsets. (E) Summary data showing the validated protocol maintains physiological T cell ratios: lymphocytes: 32.73% ± 5.95%; CD3^+^ T cells: 67.23% ± 8.21% of lymphocytes; CD4^+^/CD8^+^ ratio: 1.60 ± 0.60 (literature concordance [22–25]). Data in Subfigures (C–E) are from biologically independent mice (*n* = 3), processed individually to assess protocol robustness across individuals.

**Figure:**
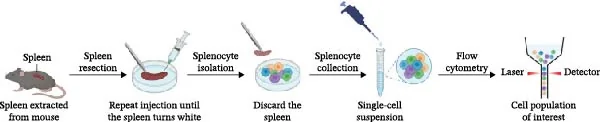
(A)

**Figure:**
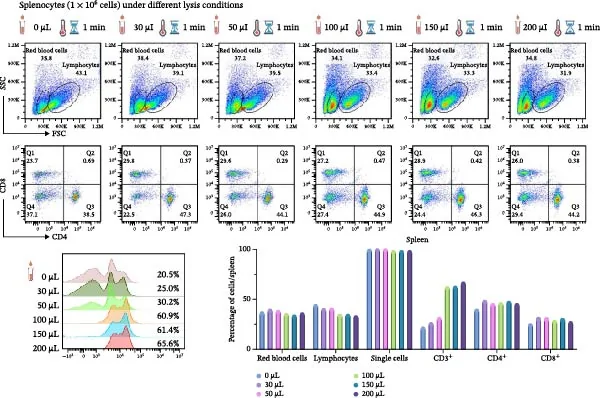
(B)

**Figure:**
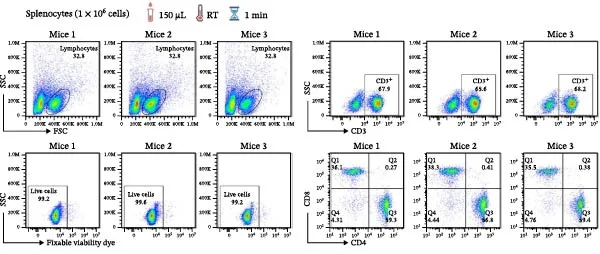
(C)

**Figure:**
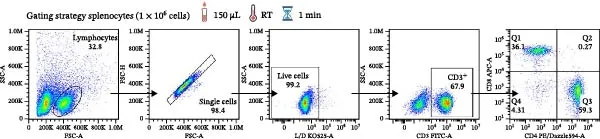
(D)

**Figure:**
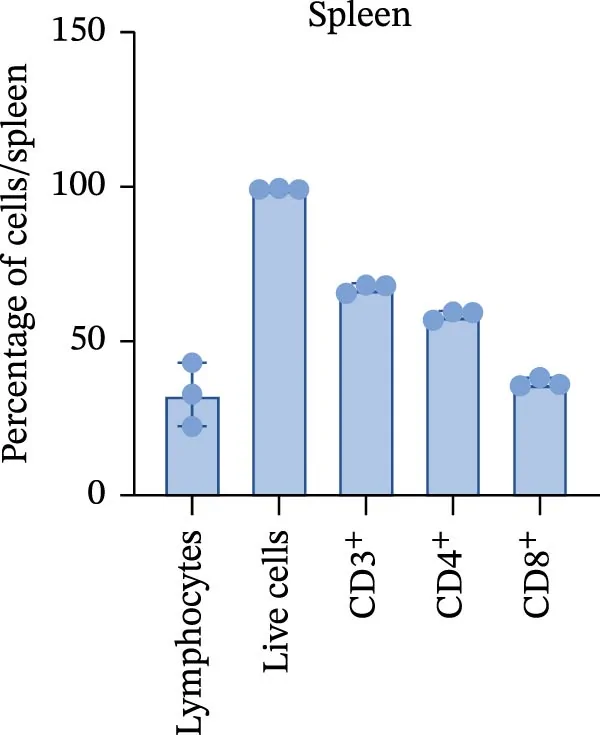
(E)

Under a lysis condition of 150 µL at room temperature for 1 min, the proportion of lymphocytes in the spleens of three mice was found to be 32.73% ± 5.95%, with CD3^+^ cells comprising 67.23% ± 8.21% of the lymphocyte population. The percentages of CD4^+^ and CD8^+^ T cells within the CD3^+^ lymphocytes were 58.50% ± 0.85% and 36.63% ± 0.85%, respectively, resulting in a CD4 to CD8 ratio of 1.60 ± 0.60 (Figure 3C–E). These findings are consistent with those reported in the literature. Shin et al. [31] documented CD4^+^ and CD8^+^ T cell proportions of 59.02% and 40.55%, respectively, in splenic cells of mice. Similarly, Kuczynski et al. [32] reported proportions of 61.8% and 33.4%, while Zamkova et al. [33] observed 70.9% and 23.6%. Comparable ratios were also noted by Pinchuk and Filipov [25]. Intragroup reproducibility was assessed by CV analysis of CD3^+^, CD4^+^, and CD8^+^ T cell proportions. As summarized in Table 4, the CV values of splenic T cell subsets (especially CD4^+^ and CD8^+^ subsets) were low, confirming that the optimized protocol yields highly consistent results even with three mice per experimental condition and supporting its reliability for splenic immunoprofiling.

Consequently, when utilizing BD lysing solution to lyse splenic samples, it is recommended that 100–200 µL of lysis solution be added per 1 × 10^6^ cells, with the samples then lysed in the dark at room temperature for 1 min. Transitioning from pooled‐sample screening (Figure 3B) to biological validation in individual subjects (Figure 3C–E), we demonstrate that the 150 μL protocol delivers both reagent economy and data robustness—essential for splenic immunoprofiling under resource constraints.

### 3.3. Consistent and Efficient Lysis of 1–5 × 10^6^ Lung Cells From Enzymatic Dissociation Using 150 μL BD Lysing Solution at Room Temperature for 1 min

Currently, enzymatic dissociation is widely used to prepare single‐cell suspensions from lung tissue (Figure 4A). Since lung tissue contains a substantial number of RBCs, erythrocyte lysis is necessary prior to flow cytometry analysis. Following the preparation of lung cell suspensions, 1 × 10^6^ cells were aliquoted per tube, and 30, 50, 100, or 150 µL of 1 × BD erythrocyte lysis buffer was added for lysis. The effects of different lysis conditions were explored using flow cytometry. Among all four conditions, the most distinct lymphocyte clustering was observed at 150 µL, followed by 100 µL (Figure 4B,C).

Figure 4Ratio‐driven volumetric titration identifies 150 μL BD lysis (1 min, RT) for universal erythrocyte clearance across enzymatic lung dissociates (1–5 × 10^6^ cells). (A) Schematic workflow of enzymatic dissociation for murine lung singlecell suspensions prepared for flow cytometry, created in BioRender. Pau, C. (2026) https://BioRender.com/mpez99o. (B, C) Titration screening. Representative flow cytometry plots (B) and quantification (C) demonstrate that 150 μL BD lysing solution (1 min, RT) maintains optimal lymphocyte gating integrity across scaled cell densities (1/3/5 × 10^6^ cells), while subvolumes (30–100 μL) show residual erythrocyte contamination. Data in Subfigures (B, C) are from technical replicates (*n* = 3 per condition) using cells pooled from three female C57BL/6J mice (adjusted to 1 × 10^7^/mL in PBS). (D–F) Protocol validation. (D) Gating strategy identifies CD45^+^ lymphocytic population. (E) Representative plots after 150 μL BD lysis at RT for 1 min. (F) Summary data showing conserved immune profiles: CD3^+^ T cells account for 96.57% ± 1.35% of CD45^+^ lymphocytes, with physiological CD4^+^/CD8^+^ distribution (CD4^+^: 58.50% ± 1.44%; CD8^+^: 39.23% ± 1.12%). Data in Subfigures (D–F) are from biologically independent mice (*n* = 3), processed individually to assess protocol robustness across individuals.

**Figure:**
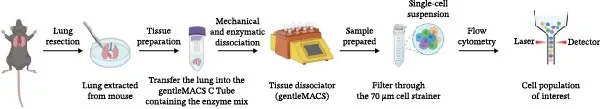
(A)

**Figure:**
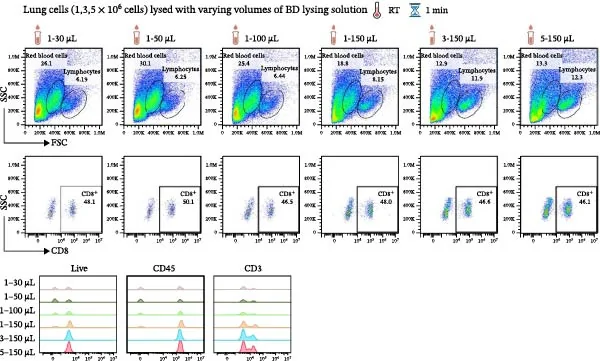
(B)

**Figure:**
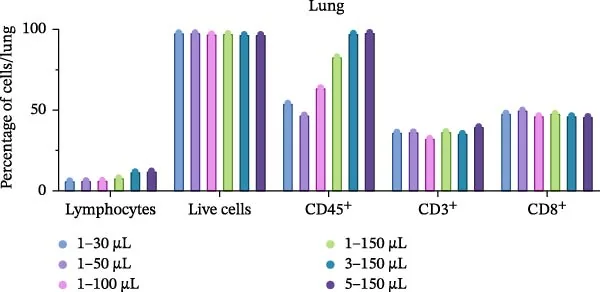
(C)

**Figure:**
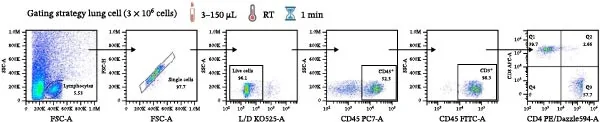
(D)

**Figure:**
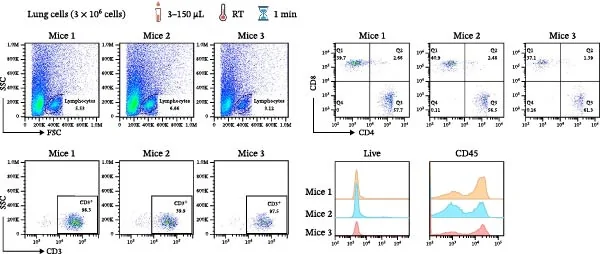
(E)

**Figure:**
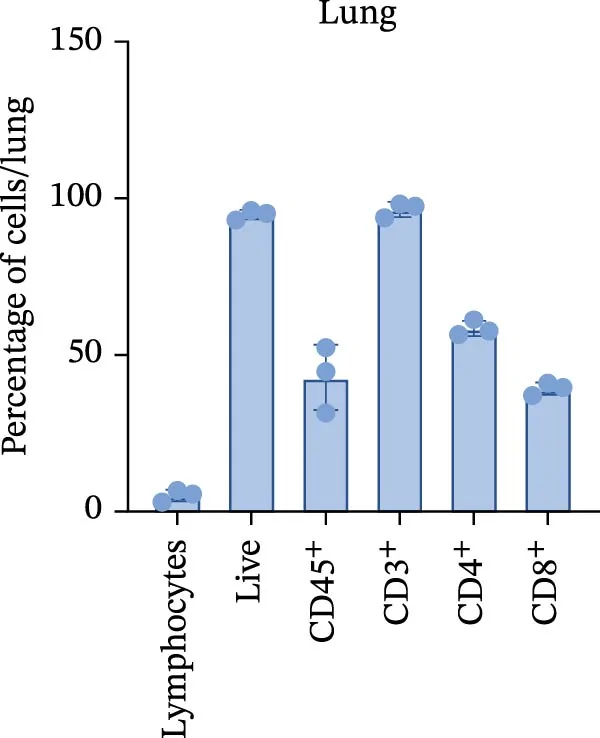
(F)

Given the relatively low proportion of lymphocytes in lung tissue, it may be necessary to increase the sample size to obtain sufficient lymphocyte numbers for analysis. Upon increasing the cell count to 3 × 10^6^ and 5 × 10^6^ cells per tube, it was found that 150 µL of 1 × BD erythrocyte lysis buffer still provided effective lysis, with clear lymphocyte clustering (Figure 4B,C). Consequently, for lung tissue samples containing 1–5 × 10^6^ cells, it is recommended that 150 µL of 1 × BD erythrocyte lysis buffer be added, thoroughly mixed, and lysed in the dark at room temperature for 1 min. The analysis of lung tissue from three mice revealed that CD45^+^ cells constituted 42.87% ± 6.05% of live cells, with CD3^+^ cells accounting for 96.57% ± 1.35% of the CD45^+^ lymphocyte population. The percentages of CD4^+^ and CD8^+^ T cells within the CD3^+^ T lymphocytes were 58.50% ± 1.44% and 39.23% ± 1.12%, respectively (Figure 4D–F). Cell yield and viability of lung cells obtained by enzymatic dissociation are detailed in Supporting Information 2: Table S1. The intragroup variability of the optimized protocol was evaluated by CV calculation of CD3^+^, CD4^+^, and CD8^+^ T cell proportions. As shown in Table 4, low CV values were observed for all lung T cell subsets, demonstrating that the protocol maintains good consistency across three biological replicates and can stably support T cell analysis in lung tissue samples.

Furthermore, we compared the results of flow cytometry analysis on lung cell suspensions prepared using tissue homogenization versus enzymatic dissociation. For the tissue homogenization procedure, 1 × 10^6^ cells were aliquoted per tube, and 0, 100, or 150 µL of 1× BD erythrocyte lysis buffer was added for lysis. Flow cytometric analysis was then conducted. In these circumstances, the lymphocyte cluster was difficult to observe (Supporting Information 3: Figure S1A).

### 3.4. Flow Cytometric Analysis of 3 × 10^6^ Mouse Bone Marrow Cells With Optimized Erythrocyte Lysis Using 100 μL BD Lysing Solution at Room Temperature for 1 min

Single‐cell suspensions from bone marrow were prepared using mechanical homogenization (Figure 5A). Cell yield and viability of bone marrow cells prepared by mechanical homogenization are presented in Supporting Information 2: Table S1. Similarly, flow cytometry analysis of T lymphocytes in bone marrow cells presents challenges akin to those encountered with lung tissue. The bone marrow contains a diverse array of cell types, with a relatively low proportion of mature lymphocytes. Furthermore, the high number of RBCs necessitates the optimization of erythrocyte lysis conditions. Given the low proportion of lymphocytes in the bone marrow, 3 × 10^6^ cells were aliquoted per tube, and 0, 100, 150, or 200 µL of 1 × BD lysing solution was added for lysis. The results demonstrated that while lymphocyte clustering was evident under the 150 and 200 µL conditions, the clusters were small and constituted a minor proportion. The 100 µL condition yielded superior results, with more pronounced lymphocyte clustering. However, the lymphocyte clusters remained smaller than those observed in the 0 µL condition (Figure 5B), indicating that the optimal lysis buffer volume may lie between 0 and 100 µL.

Figure 5Ratio‐driven volumetric titration identifies 33.3 μL/million bone marrow cells under 1‐min RT lysis for optimal lymphocyte preservation with minimal residual erythrocytes. (A) Schematic depicts mechanical dissociation protocol for murine bone marrow single‐cell suspensions. Created in BioRender. Pau, C. (2026) https://BioRender.com/mpez99o. (B, C) Titration screening. The 100 μL lysate achieves superior lymphocyte resolution against suboptimal volumes (0–75 μL showing residual erythrocytes; 200 μL causing viability loss) and enables CD45^+^/CD3^+^ clustering. (B) Representative plots. (C) Summary data demonstrating superior erythrocyte clearance at 100 μL (erythrocyte proportion: 9.75%) versus ≥ 18.1% erythrocyte proportion at ≤ 75 μL conditions, along with higher percentages of CD45^+^ cells, CD3^+^ cells, CD8^+^ cells (as a proportion of total cells), and higher CD8^+^ cell counts compared to ≤ 75 μL conditions. Data in Subfigures (B, C) are from technical replicates (*n* = 3 per condition) using pooled bone marrow from three female C57BL/6J mice. (D, E) Protocol validation. (D) Representative plots after 100 μL BD lysis at RT for 1 min. (E) Summary quantification confirming the validated protocol maintains physiological hematopoietic profiles: CD3^+^ T cells constitute 21.80% ± 3.26% of CD45^+^ lymphocytes, with a CD4^+^/CD8^+^ ratio of 0.63 ± 0.05. Data in Subfigures (D, E) are from biologically independent mice (*n* = 3), processed individually to assess protocol robustness across individuals.

**Figure:**
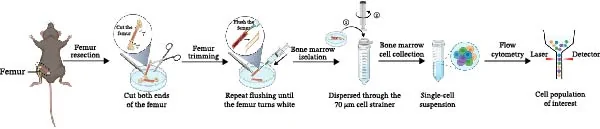
(A)

**Figure:**
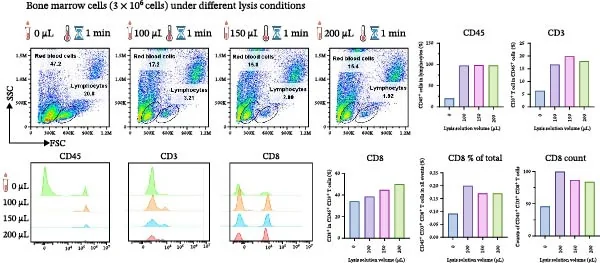
(B)

**Figure:**
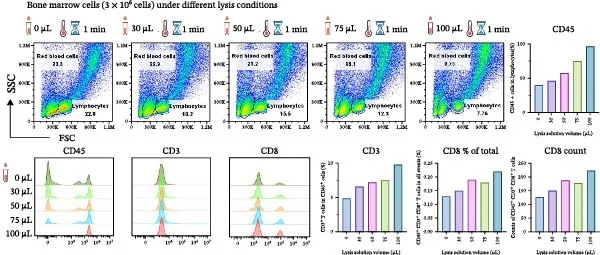
(C)

**Figure:**
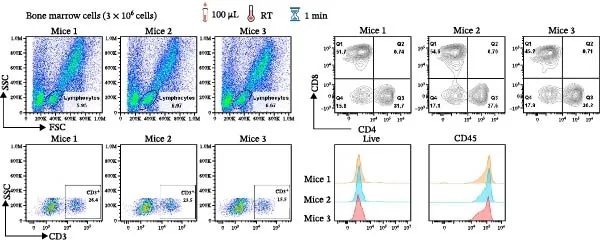
(D)

**Figure:**
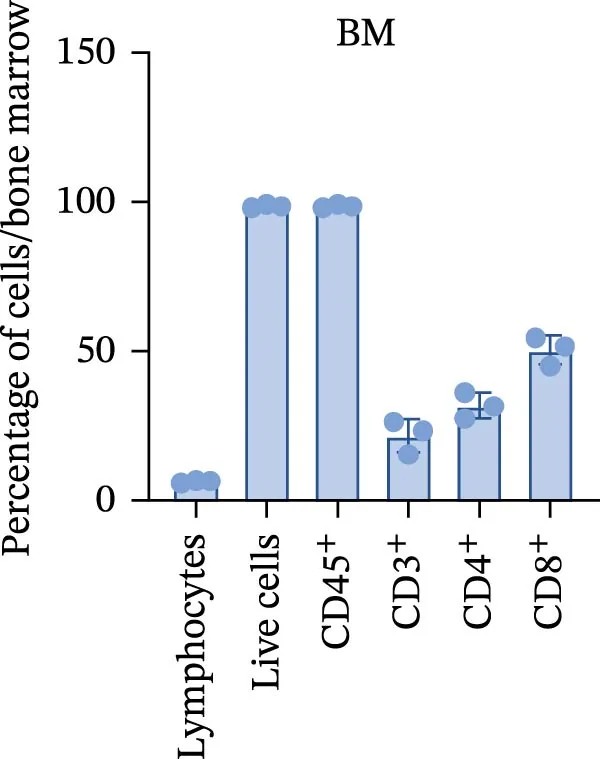
(E)

Therefore, we further explored the lysis conditions using 0, 30, 50, 75, and 100 µL of 1 × BD erythrocyte lysis buffer. The results showed that the 100 µL condition still provided the best outcomes, with the most distinct lymphocyte clustering. The percentages of CD45^+^ cells, CD3^+^ cells within CD45^+^ cells, and CD8^+^ cells within CD3^+^ cells were the highest among the five conditions. In contrast, the 30, 50, and 75 µL conditions resulted in insufficient lysis, with poor separation of lymphocytes from RBCs and cell debris (Figure 5C). Therefore, for 3 × 10^6^ bone marrow cells, we recommend adding 100 µL of 1 × BD lysing solution, mixing thoroughly, and lysing in the dark at room temperature for 1 min.

Using this condition, the analysis of bone marrow from three mice revealed that lymphocytes constituted 6.53% ± 0.30% of the total cells, with CD3^+^ cells comprising 21.80% ± 3.26% of the CD45^+^ lymphocyte population. The percentages of CD4^+^ and CD8^+^ T cells within the CD3^+^ T lymphocytes were 31.83% ± 2.48% and 50.50% ± 2.78%, respectively. The proportions of CD4^+^ and CD8^+^ T cells within the bone marrow cells were 57.34% ± 4.60% and 50.50% ± 2.78%, respectively (Figure 5D,E and Supporting Information 3: Figure S1B). Reanalyzing the results using the gating strategy described by Hensel et al. [34], we found that the percentages of CD4^+^ cells within live bone marrow cells were 3.19% ± 0.18% and 4.98% ± 1.08%, aligning with the reported ranges for healthy C57BL/6 mice bone marrow, which are 3.15%−7.76% for CD4^+^ T cells and 4.28%−5.60% for CD8^+^ T cells [34].

Intragroup consistency of the optimized bone marrow lysis protocol was verified by CV analysis of CD3^+^, CD4^+^, and CD8^+^ T cell proportions (Table 4). Although the CV value of CD3^+^ T cells was relatively higher, this may be related to biological differences among individuals, technical variability in flow cytometry experiments, or individual sensitivity to the lysis buffer; nonetheless, the CV values of CD4^+^ and CD8^+^ subsets remained acceptable. These results confirm that the optimized protocol can generate reliable data for bone marrow T cell analysis with three biological replicates.

### 3.5. Comparison of Reproducibility Between the Optimized Protocol and Traditional Methods

To comprehensively evaluate the intragroup reproducibility of the optimized erythrocyte lysis protocol across four target tissues (peripheral blood, spleen, lung, and bone marrow), we summarized the CV values of CD3^+^, CD4^+^, and CD8^+^ T cell proportions in Table 4. Most T cell subsets across tissues exhibited low CV values (1.40%−8.41%), except for CD3^+^ T cells in spleen (12.21%) and bone marrow (14.95%). This relatively higher variability may be attributed to inherent biological differences among individual mice, technical variations in flow cytometry (e.g., tissue dissociation efficiency, antibody staining uniformity, and gating judgments for low‐abundance CD3^+^ populations), and individual differences in CD3^+^ T cell sensitivity to lysis buffer. Collectively, these results indicate that the optimized protocol can yield consistent results across multiple murine immune compartments, supporting its potential reliability for T cell analysis with three biological replicates.

To further explore the reproducibility of the proposed protocol in comparison with existing methods, we compared its T cell subset CV values with those of traditional ‘fixed volume/time’‐dependent lysis methods from five independent studies (Table 4). Traditional protocols were found to exhibit substantially higher CV values (4.81%−66.86%) across different tissues and subsets. For example, Litvinova et al. [26] reported a CD3^+^ T cell CV of approximately 66.86% in peripheral blood, Magee et al. [28] reported a CD4^+^ T cell CV range of approximately 23.53%−43.75% in spleen, and Jiang et al. [27] reported a CD8^+^ T cell CV of approximately 20.74% in peripheral blood. In comparison, our optimized protocol maintained relatively low CV values (1.40%−14.95%) across the four tissues. Even the subsets with relatively higher CVs (spleen and bone marrow CD3^+^ T cells) were lower than most CV values of traditional methods, and most CD4^+^ and CD8^+^ subsets had CVs below 9%. These results suggest that our volume ratio–based standardized protocol may have more favorable reproducibility compared to traditional methods.

In addition, to validate the reduction of operator dependency, three independent operators each processed one mouse sample following the standardized SOP. Low CV values of T cell subsets were observed across operators (e.g., peripheral blood CD8^+^ T cell CV = 1.56% and spleen CD8^+^ T cell CV = 2.32%), which were comparable to intragroup CVs of a single operator. This observation indicates that the protocol is conducive to minimizing operator‐dependent variability.

## 4. Discussion

Flow cytometry of peripheral blood, spleen, lung, and bone marrow in mice is essential in immunology. Reliable and reproducible protocols are critical for analyzing the immune system and studying immune disorders, providing comprehensive data on T lymphocyte development, maturation, differentiation, and tissue residency. Erythrocyte lysis is a pivotal step; insufficient lysis can obscure lymphocyte separation, while excessive lysis can damage lymphocytes. The efficacy of lysis solutions varies significantly between brands, requiring researchers to optimize conditions. This study outlines detailed protocols for sampling, single‐cell preparation, erythrocyte lysis, and flow cytometry and compares different lysis solutions to identify optimal conditions for consistent lymphocyte analysis.

Current protocols for flow cytometry in murine blood, spleen, bone, and bone marrow typically involve erythrocyte lysis prior to cell counting to minimize the impact of erythrocytes on the counting results [9, 10, 35, 36]. However, the lysis process presents several challenges. Insufficient lysis can lead to excessive residual RBCs, which interfere with flow cytometry results. In such cases, repeated lysis may be necessary to further reduce RBC interference. However, this can cause additional damage to lymphocytes, making sample recovery difficult, particularly for precious samples. To mitigate this issue, we perform cell counting prior to erythrocyte lysis using a nucleated cell counting solution to exclude RBC interference on cell counting results. Based on these counts, only a small portion of the sample is aliquoted for erythrocyte lysis. This approach ensures that sufficient sample remains for repeat or other experiments if needed and allows for the comparison of different lysis solutions.

The spleen contains various cell types, including RBCs, lymphocytes, endothelial cells, reticular cells, smooth muscle cells, myofibroblasts, macrophages, dendritic cells, and neutrophils. Traditional splenic cell suspension preparation primarily uses mechanical grinding. Our protocol, however, utilizes repeated syringe injections to flush out free RBCs and lymphocytes. This method is simpler and, while yielding fewer cells than mechanical grinding, provides enough for downstream flow cytometry. Its main advantage is minimizing mechanical damage to lymphocytes and reducing contamination from other cell types, making it ideal for experiments requiring high‐quality lymphocytes.

We present a simple and effective flow cytometry method for lung and bone marrow samples that better preserves lymphocytes, allowing accurate analysis of lymphocyte percentages. Christofides et al. [9] identified monocytes, neutrophils, NK cells, polymorphonuclear neutrophils, and eosinophils in lung tissue using enzymatic digestion. Similarly, Singer et al. [37] identified epithelial cells, endothelial cells, hematopoietic cells, and lineage‐negative cells. However, identifying lymphocytes in lung and bone marrow is challenging due to the low proportion of lymphocytes and the presence of other cell types. Direct analysis requires large sample volumes and extended acquisition times. For more effective analysis, lymphocytes can be separated and enriched using lymphocyte isolation media before flow cytometry.

This study has inherent limitations that should be acknowledged. The high‐throughput comparative design necessitated pooling samples from three mice per experimental condition, resulting in a relatively small sample size that precluded rigorous formal statistical analysis. To address this, we adopted descriptive statistical approaches and explicitly reported all sample sizes to ensure research transparency, thereby establishing a robust baseline for subsequent hypothesis‐driven investigations. We further acknowledge that the use of three mice per group may constrain the generalizability of the optimized protocol. As appropriately highlighted by the reviewer, increasing the number of biological replicates (e.g., expanding to five mice per experimental condition) would significantly enhance the robustness and statistical validity of the findings. This constitutes a key focus of our future work, where we aim to validate the optimized protocol in expanded sample cohorts to confirm its applicability across a broader spectrum of murine models and experimental contexts.

Notwithstanding this limitation, the current sample size is sufficient to support the core objective of this study—developing a standardized erythrocyte lysis framework for reliable and reproducible T cell analysis. The results revealed low CV values for CD8^+^ T cell proportions across all four target tissues: approximately 1.56% in peripheral blood (38.40% ± 0.60%), approximately 2.32% in spleen (36.63% ± 0.85%), approximately 2.85% in lung (39.23% ± 1.12%), and approximately 5.50% in bone marrow (50.50% ± 2.78%). These low CV values demonstrate that the optimized protocol yields highly consistent intragroup data even with three biological replicates. Furthermore, the distribution patterns of T cell subsets (e.g., CD4^+^/CD8^+^ ratio) in each tissue are consistent with well‐documented phenotypic characteristics of the murine immune system, which further corroborates the biological relevance and validity of our findings. Collectively, the standardized protocol established herein provides a reliable foundation for reproducible T cell analysis across multiple murine immune compartments, and future studies with expanded sample sizes will further strengthen its scientific rigor and translational potential.

While the present work focused on optimizing erythrocyte lysis for T cell analysis, the key innovation lies in the development of a ratio‐driven volumetric precision method. By scaling the lysis buffer volume to the nucleated cell count and processing only a small aliquot of the sample, the majority of the original specimen is preserved. Importantly, we observed differential sensitivity of various immune cell types to commercial lysis buffers (Figure 2B), emphasizing the necessity of exploring and optimizing cell‐type‐specific lysis conditions. This experimental platform thus facilitates the systematic optimization of lysis protocols for diverse immune cell populations (e.g., B cells, NK cells, and macrophages) derived from the same specimen, ultimately achieving a superior balance between efficient erythrocyte clearance and immunocyte preservation. Another notable limitation is the absence of a true “0‐min” lysis control. This control was omitted due to the high concentration of intact erythrocytes in unlysed blood samples, which poses a risk of cytometer clogging and impedes accurate lymphocyte detection. Consequently, our study compared the relative lysis efficiency and lymphocyte preservation across subsequent time points. Future investigations could incorporate a minimally lysed, highly diluted sample acquired immediately after buffer addition to establish a more robust kinetic baseline.

Notably, our standardized erythrocyte lysis framework holds translational value for clinical research on immune‐related diseases. Accumulating evidence highlights the critical interplay between the heart, spleen, and bone marrow in the pathogenesis of diseases such as heart failure, where splenic function and bone marrow‐derived immune cells serve as key prognostic indicators and therapeutic targets [38, 39]. Our protocol enables robust and reproducible T cell analysis across these key immune compartments in murine models, which provides a reliable methodological basis for preclinical studies investigating cross‐system immune mechanisms. This, in turn, facilitates the translation of preclinical findings to clinical practice, supporting the development of novel diagnostic and therapeutic strategies for diseases involving multiorgan immune dysregulation.

In summary, this study establishes a paradigm‐shifting lysis framework through three synergistic innovations: tissue‐adapted volumetric scaling, operator‐independent timed lysis, and erythrocyte‐exclusion counting cell census. These advances, combined with hydrodynamic cell isolation refinements, collectively overcome the reproducibility crisis in multitissue T cell analysis. By decoupling experimental outcomes from technical variability, our workflow sets a new standard for cross‐tissue cytometry while offering a blueprint for precision cell processing in translational immunology.

## 5. Conclusions

This study establishes a standardized and robust framework for erythrocyte lysis, enabling reliable and reproducible T cell analysis across diverse murine immune compartments—peripheral blood, spleen, lung, and bone marrow. Critically, we identified the BD lysing solution as a superior and unified buffer providing an optimal balance between efficient erythrocyte removal and lymphocyte integrity preservation. The core innovation lies in replacing inconsistent fixed volume or variable duration methods with ratio‐driven volumetric precision, where lysis buffer volume is dynamically scaled to precounted nucleated cells using an acetic acid/methylene blue census, coupled with a strict 1‐min lysis duration to minimize operator‐induced variability. Tissue‐specific volumetric optimizations (150 μL/million splenocytes; 30–150 μL/million lung cells; 33.3 μL/million bone marrow cells) resolved unique barriers in each tissue, yielding high‐fidelity T cell frequencies and subset distributions (CD4/CD8 ratios). Collectively, these validated protocols provide an essential universal standard, ensuring operator‐independent accuracy crucial for comparative immunology studies and enhancing the reproducibility of translational research in murine models.

## Author Contributions

Run‐Ze Li conceptualized and designed the study, secured funding, administered the project, supervised all research stages, reviewed and edited the manuscript, and approved the final version. Wei‐Qian Bao developed methodology, implemented research protocols, curated data, and drafted the manuscript. Meng‐Lin Jiang and Nan‐Jie Zhou designed and executed experiments and acquired data. Li‐Rong Lian, Shu‐qin Li, Bi‐qing Huang, and Tu‐Liang Liang contributed to experimental execution and data collection. Gang‐Yuan Ma provided technical support for experiments, maintained equipment, and assisted in data analysis. Cui‐Fen Zhang and Ying Chen managed sample collection/reagents and supported experimental operations/data recording. Xiao Shu assisted with data visualization/formatting. Liang Liu and Hu‐Dan Pan coordinated resources and project timelines (Liang Liu with external communications and Hu‐Dan Pan with ethics oversight and data security).

## Funding

This work was financially supported by a grant from the National Natural Science Foundation of China (Grant 82474267), the Noncommunicable Chronic Diseases‐National Science and Technology Major Project (Grant 2024ZD0521405), the Major Project of Guangzhou National Laboratory (Grant GZNL2023A02009), the Natural Science Foundation of Guangdong Province, China (Grant 2026B1515020086), the “Pearl River Talent Plan” Innovation and Entrepreneurship Team Introduction Projects of Guangdong Province (Grant 2023ZT10Y053), the Traditional Chinese Medicine Bureau of Guangdong Province Project (Grant 20241112), the State Key Laboratory of Traditional Chinese Medicine Syndrome (Grant QZ2023ZZ11), and the Chinese Medicine Guangdong Laboratory (Grants HQL2024PZ005 and HQL2025CY007).

## Disclosure

All authors reviewed and approved the final manuscript.

## Conflicts of Interest

The authors declare no conflicts of interest.

## Supporting Information

Additional supporting information can be found online in the Supporting Information section.

## Supporting information


**Supporting Information 1** Standard operating procedure (SOP): This SOP details all critical steps of the tissue‐specific erythrocyte lysis protocols established in this study. Standardized operational guidelines ensure protocol reproducibility, minimize interoperator technical variations, and support robust T cell analysis.

**Supporting Information 2** Table S1. Cell yield and viability data obtained by corresponding processing methods for lung and bone marrow tissues. Table S1 records the cell yield and viability data obtained by corresponding processing methods for lung and bone marrow tissues. Its notes detail the raw data of three biological replicates, clarify the data sources (actual experimental measurements), detection methods (flow cytometry for viability), and normalization method (per organ), ensuring the transparency and traceability of the data. This table provides direct evidence for the reproducibility and practicality of the proposed enzymatic dissociation method.

**Supporting Information 3** Figure S1. Mechanical homogenization impedes pulmonary lymphocyte resolution and gating strategy for the flow cytometric analysis of bone marrow cells. (A) Impact of dissociation method on resolution. Representative flow cytometry plots of mechanically homogenized lung cells lysed with 0–150 μL BD buffer (1 min, RT) show obscured lymphocyte clustering compared to enzymatic dissociation (cf. Figure 4B). Data are from technical replicates (*n* = 3 per condition) using pooled lung cells from three female C57BL/6J mice. (B) Bone marrow gating strategy. Gating strategy for the flow cytometric analysis of bone marrow cells (3 × 10^6^ cells) lysed with 100 μL BD lysing solution (RT, 1 min). Data are from biologically independent mice (*n* = 3).

## Data Availability

The data that support the findings of this study are available from the corresponding author upon reasonable request.
