# Supplementary material for: Optimization of Erythrocyte Lysis Protocols for Robust and Reproducible T Cell Analysis in Murine Peripheral Blood, Spleen, Lung, and Bone Marrow
Source: J Immunol Res. 2026 Apr 7;2026:3335332. doi: 10.1155/jimr/3335332 (PMC13581057; doi:10.1155/jimr/3335332)
Supplement: Supplementary file 1 [file JIMR-2026-3335332-s003.pdf]

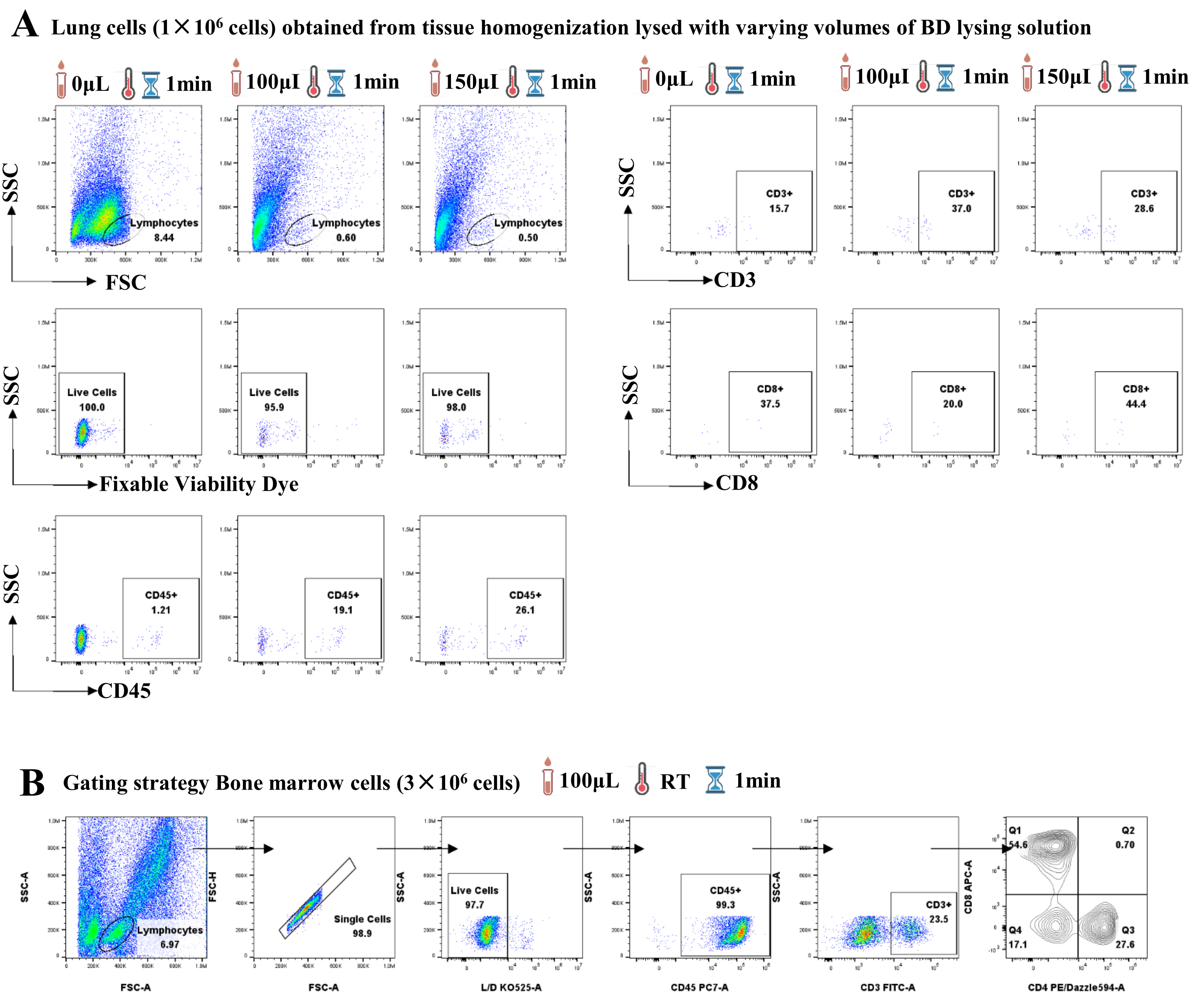

Supplemental Figure S1. Mechanical homogenization impedes pulmonary lymphocyte resolution and gating strategy for the flow cytometric analysis of bone marrow cells.

(A) Impact of Dissociation Method on Resolution. Representative flow cytometry plots of mechanically homogenized lung cells lysed with 0-150μL BD buffer (1 min, RT) show obscured lymphocyte clustering compared to enzymatic dissociation (cf. Figure 4B). *Data are from technical replicates (n = 3 per condition) using pooled lung cells from three female C57BL/6J mice.*

(B) Bone Marrow Gating Strategy. Gating strategy for the flow cytometric analysis of bone marrow cells (3×10⁶ cells) lysed with 100 μL BD lysing solution (RT, 1 min). *Data are from biologically independent mice (n = 3).*
