# Supplementary material for: Optimization of Erythrocyte Lysis Protocols for Robust and Reproducible T Cell Analysis in Murine Peripheral Blood, Spleen, Lung, and Bone Marrow
Source: J Immunol Res. 2026 Apr 7;2026:3335332. doi: 10.1155/jimr/3335332 (PMC13581057; doi:10.1155/jimr/3335332)
Supplement: Supplementary file 2 [file JIMR-2026-3335332-s001.docx]

**Standard Operating Procedure (SOP)**

- **Main reagents and materials**

**Table 1: Main reagents and materials.**

| **Reagent or Resource** | **Source** | **Identifier** |
| --- | --- | --- |
| Fixable Viability Stain 620 | BD | Cat#564996 |
| Ghost Dye™ Violet 540 Fixable Viability Dye | TONBO | Cat#72086 |
| anti - CD45 antibody | BD | Cat#552848 |
| anti - CD3 antibody | BioLegend | Cat#100234 |
| anti - CD3 antibody | BioLegend | Cat#100204 |
| anti - CD4 antibody | BD | Cat#553051 |
| anti - CD4 antibody | BioLegend | Cat#100456 |
| anti - CD8a antibody | BioLegend | Cat#100706 |
| anti - CD8a antibody | BioLegend | Cat#100712 |
| Lysing Solution 10X Concentrate | BD | Cat#349202 |
| RBC Lysis Buffer (10X) | BioLegend | Cat#420301 |
| Red Blood Cell Lysis Buffer | Biosharp | Cat#BL503B |
| 3% Acetic Acid with Methylene Blue | Stemcell | Cat#07060 |
| Fetal Bovine Serum (FBS) | ExCell | Cat#FSP500 |
| Albumin from bovine serum | Macklin | Cat#B917420 - 200g |
| Phosphate Buffer Saline (PBS) | Solarbio | Cat#G0002 |
| MACS Tissue Storage Solution | Miltenyi | Cat#130 - 100 - 008 |
| Lung Dissociation Kit, mouse | Miltenyi | Cat#130 - 095 - 927 |
| gentleMACS 25C Tubes C | Miltenyi | Cat#130 - 093 - 237 |
| 70μm cell strainers | Biosharp | Cat#BS - 70 - XBS - N1 |
| Heparin sodium anticoagulant tube 1.5ml | Solarbio | Cat#YA1471 |
| Isoflurane anesthetic | RWD | Cat#R510 - 22 - 10 |

**Table 2: Composition of buffers for erythrocyte lysis and cell staining.**

| **Buffer Type** | **Composition (units included in description)** |
| --- | --- |
| PEB buffer | PBS + 2% FBS+1mM EDTA or PBS+0.5% BSA+1mM EDTA |
| Cell Staining Buffer | PBS + 2% FBS |
| 1× lysing solution | Lysing solution 10× concentrate diluted 10× in purified deionized water |

**Table 3: Usage of monoclonal antibodies.**

| **Antigen** | **Clone** | **Fluorochrome** | **Dilution** | **Surface/intracellular** |
| --- | --- | --- | --- | --- |
| Fixable Viability Stain 620 | / | FVS620 | 1:1000 | Surface |
| Ghost Dye™ Violet 540 Fixable Viability Dye | / | Violet 540 | 1:5000 | Surface |
| CD45 | 30-F11 | PE-Cy7 | 3:1000 | Surface |
| CD3 | 17A2 | BV510 | 1:200 | Surface |
| CD3 | 17A2 | FITC | 1:500 | Surface |
| CD4 | RM4-5 | APC | 1:800 | Surface |
| CD4 | GK1.5 | PE/Dazzle™ 594 | 1:800 | Surface |
| CD8a | 53-6.7 | FITC | 1:500 | Surface |
| CD8a | 53-6.7 | APC | 1:800 | Surface |

- **Mouse Anesthesia and Terminal Blood Collection**

1. Animal Preparation: Confirm the use of female C57BL/6J mice, 6–8 weeks old, SPF grade.

2. Anesthesia Induction: Administer tribromoethanol via intraperitoneal injection at a dose of 250 mg/kg.

3. Anesthesia Depth Verification: Ensure loss of pedal withdrawal reflex and maintain respiratory rate between 80–120 breaths per minute before proceeding.

4. Terminal Blood Collection: Perform cardiac puncture under deep anesthesia to collect terminal blood samples.

5. Euthanasia: Following blood collection, promptly perform cervical dislocation as a secondary method of euthanasia.

**Note:** All procedures must be approved by the Animal Ethics Committee. Operations must be conducted by trained personnel in a barrier environment.

- **Flow cytometric analysis of T lymphocytes in mouse peripheral blood**

1. Sample preparation: Whole blood was collected from anesthetized mice using heparin sodium anticoagulation. A 50 μL aliquot of heparin sodium-anticoagulated whole blood was transferred into pre-chilled 1.5 mL microcentrifuge tubes for immediate flow cytometric analysis.

2. Red blood cell lysis: Prepare 1×BD lysis solution (Tables 1 and 2). Add 500 µL 1×BD lysis solution and mix well, incubate for 1min at room temperature and protect from light. Immediately add 500 µL of pre-cooled cell staining buffer or PEB solution (Table 2) and mix thoroughly to terminate the lysis. Centrifuge at 350g for 5min at 4℃ and discard the supernatant.

3. Wash twice: Resuspend the cell pellet in 1 mL of PBS, then centrifuge at 300g for 3 minutes at 4°C, followed by discarding the supernatant. Repeat this step once. After the second centrifugation, leave approximately 100 µL of volume.

4. Viability Dye Staining: Prepare a dilution of the viability dye in PBS (Table 2). Add 1 ml of the dye dilution and vortex immediately. Incubate the mixture for 10-15 minutes at room temperature protected from light (follow the specific instructions for the viability dye used). After incubation, centrifuge immediately at 300g for 3 minutes at 4°C.

5. Wash once: Resuspend the cell pellet in 1 mL of PBS, then centrifuge at 300g for 3 minutes at 4°C, followed by discarding the supernatant. After the second centrifugation, leave approximately 100 µL of volume.

6. Extracellular staining: Prepare dilutions of surface antibodies as outlined in Table 3. Add the appropriate dilution to each respective single staining tube and sample tube. Thoroughly mix and incubate the tubes on ice for 20 minutes, protecting them from light.

**Note:** Prior to experimental use, it is advisable to optimize the concentration of each antibody through titration to achieve optimal staining efficacy and minimize costs.

7. Wash twice: Resuspend the cell pellet in 1 mL of PBS, then centrifuge at 300g for 3 minutes at 4°C, followed by discarding the supernatant. Repeat this step once.

8. Resuspend cells: Resuspend the cell pellet in 800 µL of cell staining buffer (Table 2), then place the suspension on ice, shielded from light, in preparation for flow cytometry analysis.

9. Flow cytometry analysis: Record at least 1×10^4^ events per sample on the flow cytometer.

**Note:** Adjust the number of recorded events as needed. Modify the flow staining protocol for intracellular or intranuclear antibody staining based on experimental requirements.

- **Flow cytometric analysis of T lymphocytes in mouse spleen**

1. Splenocyte suspension preparation: Carefully excise the mouse spleen and rinse it in ice-cold PBS. Then, place it in a culture dish containing 2 ml of ice-cold PBS. Hold the spleen with forceps in the left hand and a 1 mL syringe in the right hand. Aspirate PBS from the culture dish using the syringe, and then carefully insert the syringe needle into the spleen tissue for injection. Observe swelling of the spleen and red liquid oozing out during injection. Repeat the injection several times until the spleen turns from dark red to light red. Rinse the dish with 1 mL of PBS and collected, and a total of 3 mL of splenocyte suspension was obtained (Figure 3A). After thorough mixing, take 20 µL of cell suspension and mix it with 20 µL of 3% acetic acid with methylene blue (Table 1) for cell counting. Transfer 1×10^6^ cells to a 1.5 mL Eppendorf tube for subsequent flow cytometry.

**Note:** The injection of splenic tissue using a syringe is proposed as a novel method for isolating splenic cells in this study. This approach is anticipated to result in less damage to splenocytes compared to conventional tissue grinding methods. Preliminary results indicate that this technique yields approximately 3×10^7^ nucleated cells per spleen, which is deemed suitable for flow analysis, albeit yielding a lower cell count compared to the grinding method.

2. Wash once: Resuspend the cell pellet in 1 mL of PBS, then centrifuge at 350g for 5 minutes at 4°C. After centrifugation, carefully aspirate and discard the supernatant to minimize the remaining liquid.

**Note:** Ensure thorough aspiration of the supernatant to minimize residual liquid, as incomplete removal can impact the efficacy of subsequent red blood cell lysis, potentially leading to incomplete lysis due to interference from residual liquid.

3. Red blood cell lysis: Prepare 1×BD lysis solution (Tables 1 and 2). Add 100-200 µL 1×BD lysis solution and mix well, incubate for 1min at room temperature and protect from light. Immediately add 1 mL of pre-cooled cell staining buffer or PEB solution (Table 2) and mix thoroughly to terminate the lysis. Centrifuge at 350g for 5min at 4℃ and discard the supernatant.

4. Wash twice: Resuspend the cell pellet in 1 mL of PBS, then centrifuge at 300g for 3 minutes at 4°C, followed by discarding the supernatant. Repeat this step once. After the second centrifugation, leave approximately 100 µL of volume.

5. Flow cytometry: Perform flow cytometry as described for the analysis of T lymphocytes in mouse peripheral blood, recording at least 1×10⁴ events per sample.

- **Flow cytometric analysis of T lymphocytes in mouse lung**

1. Lung Tissue Removal: Carefully remove the heart, then separate the lung from the surrounding tissue. Rinse with PBS to remove excess blood and place the lung in cold PBS on ice for temporary storage (Figure 4A).

**Note:** To avoid interference from circulating lymphocytes in the blood, inject 10 mL of cold PBS directly into the right ventricle using an 18-21G needle until the lungs turn completely white before removing the heart and lung tissue. Ensure all adjacent connective tissue is removed from the lungs before preparing single-cell suspensions, as residual tissue may bias the results.

2. Lung Dissociation: Follow the instructions of the Lung Dissociation Kit, mouse (Table 1), to dissociate the lung tissue into a single-cell suspension using the gentleMACS™ Octo Dissociator with Heaters (Miltenyi, Cat# 130-096-427) (Figure 5A).

3. Filtration: Filter the dissociated single-cell suspension through a 70 μm cell strainer. Rinse the strainer by adding 2.5 mL of 1× Buffer S (refer to instruction of the Lung Dissociation Kit, mouse) (Figure 4A).

4. Centrifugation: Centrifuge at 300g for 10 minutes. Discard the supernatant and resuspend the cells in 3 mL of cell staining buffer (Table 2).

5. Cell Counting: Combine 20 µL of the cell suspension with 20 µL of 3% acetic acid containing methylene blue (Table 1) and mix thoroughly. Count the cells, then transfer 3×10^6^ cells per tube for flow cytometry.

**Note:** Given the relatively low proportion of lymphocytes in lung tissue, increasing the number of cells in the sample to 3×10^6^ cells per tube is necessary to obtain sufficient lymphocytes for analysis in this study. It is recommended to adjust the number of cells to between 1×10^6^ and 5×10^6^ cells per tube according to specific experimental requirements.

6. Wash once: Resuspend the cell pellet in 1 mL of PBS, then centrifuge at 350g for 5 minutes at 4°C. After centrifugation, carefully aspirate and discard the supernatant to minimize the remaining liquid.

**Note:** Ensure thorough aspiration of the supernatant to minimize residual liquid, as incomplete removal can impact the efficacy of subsequent red blood cell lysis, potentially leading to incomplete lysis due to interference from residual liquid.

7. Red blood cell lysis: Prepare 1×BD lysis solution (Tables 1 and 2). Add 150 µL 1×BD lysis solution and mix well, incubate for 1min at room temperature and protect from light. Immediately add 1 mL of pre-cooled cell staining buffer or PEB solution (Table 2) and mix thoroughly to terminate the lysis. Centrifuge at 350g for 5min at 4℃ and discard the supernatant.

8. Wash twice: Resuspend the cell pellet in 1 mL of PBS, then centrifuge at 300g for 3 minutes at 4°C, followed by discarding the supernatant. Repeat this step once. After the second centrifugation, leave approximately 100 µL of volume.

9. Flow cytometry: Perform flow cytometry as described for the analysis of T lymphocytes in mouse peripheral blood, recording at least 1×10^5^ events per sample.

- **Flow cytometric analysis of T lymphocytes in mouse bone marrow**

1. Femur removal: Isolate the femur from mice and remove muscles and fibrous tissues. Excess muscle tissues can be wiped off with gauzes. Store the femur in 2 mL of cold PBS on ice for temporary storage (Figure 5A).

2. Bone perfusing: Cut off both ends of the femur, retaining only the body. Place the bone upright in a 35 mm dish containing cold PBS. Using a 1 mL syringe, aspirate PBS and carefully flush out the bone marrow from one end of the femur. Repeat until the bone turns white, indicating that all bone marrow has been flushed out (Figure 5A).

3. Bone marrow cell suspension preparation: Place a 70 µm cell strainer on top of a 50 mL centrifuge tube and rinse with 1 mL of cold PBS. Transfer the bone marrow suspension into the strainer using a pipette. Rinse the dish with an additional 1 mL of PBS and collect the remaining solution into the strainer. Gently grind the remaining bone marrow tissue on the strainer mesh with the plunger of a 1 mL syringe until no clumps remain. Rinse the strainer with 2 mL of cold PBS to obtain a total of 5 mL of single-cell suspension (Figure 5A).

4. Cell counting: Combine 20 µL of the cell suspension with 20 µL of 3% acetic acid containing methylene blue (Table 1) and mix thoroughly. Count the cells, then transfer 3×10^6^ cells per tube for flow cytometry.

5. Wash once: Resuspend the cell pellet in 1 mL of PBS, then centrifuge at 350g for 5 minutes at 4°C. After centrifugation, carefully aspirate and discard the supernatant to minimize the remaining liquid.

**Note:** Ensure thorough aspiration of the supernatant to minimize residual liquid, as incomplete removal can impact the efficacy of subsequent red blood cell lysis, potentially leading to incomplete lysis due to interference from residual liquid.

6. Red blood cell lysis: Prepare 1×BD lysis solution (Tables 1 and 2). Add 100 µL 1×BD lysis solution and mix well, incubate for 1min at room temperature and protect from light. Immediately add 1 mL of pre-cooled cell staining buffer or PEB solution (Table 2) and mix thoroughly to terminate the lysis. Centrifuge at 350g for 5min at 4℃ and discard the supernatant.

7. Wash twice: Resuspend the cell pellet in 1 mL of PBS, then centrifuge at 300g for 3 minutes at 4°C, followed by discarding the supernatant. Repeat this step once. After the second centrifugation, leave approximately 100 µL of volume.

8. Flow cytometry: Perform flow cytometry as described for the analysis of T lymphocytes in mouse peripheral blood, recording at least 1×10⁴ events per sample.
