# Supplementary material for: Optimization of Erythrocyte Lysis Protocols for Robust and Reproducible T Cell Analysis in Murine Peripheral Blood, Spleen, Lung, and Bone Marrow
Source: J Immunol Res. 2026 Apr 7;2026:3335332. doi: 10.1155/jimr/3335332 (PMC13581057; doi:10.1155/jimr/3335332)
Supplement: Supplementary file 3 [file JIMR-2026-3335332-s002.docx]

**Supplemental Table S1: Cell Yield and Viability Data Obtained by Corresponding Processing Methods for Lung and Bone Marrow Tissues**

| **Tissue Type** | **Method** | **n** | **Cell Yield (cells/organ, Mean ± SD)** | **Cell Viability: Flow Cytometry (%, Mean ± SD)** |
| --- | --- | --- | --- | --- |
| Lung | Enzymatic Digestion | 3 | 2.5±0.6×10⁶ | 94.87 ± 1.42 |
| Bone Marrow | Flushing and Homogenization | 3 | 6.83± 0.41×10⁷ | 98.67 ± 0.62 |

Notes: 1. 1. All data are derived from actual experimental measurements of 3 independent biological replicates using C57BL/8J mice (n=3). The detailed raw data are as follows: Lung tissue (enzymatic digestion): cell yield per lung was 2.2×10⁶, 2.9×10⁶, 2.5×10⁶ cells, and the corresponding live cell rates were 96.1%, 95.3%, 93.2%; Bone marrow tissue (flushing and homogenization): cell yield per bilateral femur was 6.4×10⁷, 7.2×10⁷, 6.9×10⁷ cells, and the corresponding live cell rates were 98.1%, 99.3%, 98.6%. 2. Cell viability was detected by flow cytometry, ensuring the accuracy and reliability of the data. 3. Cell yield was normalized by "per organ" to standardize the data presentation, which is conducive to evaluating the reproducibility and practicality of the corresponding processing methods.
